# Emerging Views on the CTD Code

**DOI:** 10.1155/2012/347214

**Published:** 2012-02-26

**Authors:** David W. Zhang, Juan B. Rodríguez-Molina, Joshua R. Tietjen, Corey M. Nemec, Aseem Z. Ansari

**Affiliations:** ^1^Department of Biochemistry, University of Wisconsin-Madison, 433 Babcock Drive, Madison, WI 53706, USA; ^2^Genome Center of Wisconsin, University of Wisconsin-Madison, 433 Babcock Drive, Madison, WI 53706, USA

## Abstract

The C-terminal domain (CTD) of RNA polymerase II (Pol II) consists of conserved heptapeptide repeats that function as a binding platform for different protein complexes involved in transcription, RNA processing, export, and chromatin remodeling. The CTD repeats are subject to sequential waves of posttranslational modifications during specific stages of the transcription cycle. These patterned modifications have led to the postulation of the “CTD code” hypothesis, where stage-specific patterns define a spatiotemporal code that is recognized by the appropriate interacting partners. Here, we highlight the role of CTD modifications in directing transcription initiation, elongation, and termination. We examine the major readers, writers, and erasers of the CTD code and examine the relevance of describing patterns of posttranslational modifications as a “code.” Finally, we discuss major questions regarding the function of the newly discovered CTD modifications and the fundamental insights into transcription regulation that will necessarily emerge upon addressing those challenges.

## 1. Introduction

The transcription of DNA to RNA in eukaryotes is catalyzed by three structurally related RNA polymerases, with each acting on a different class of genes [1]. RNA polymerase I synthesizes most of the ribosomal RNA (rRNA) subunits while RNA polymerase III synthesizes tRNAs, 5S rRNA, and other small RNAs [2–4]. These two polymerases account for 75% and 15% of transcription in the cell, respectively [5]. However, the most studied polymerase is RNA Polymerase II (Pol II), which is responsible for the transcription of protein-coding genes, small nuclear RNA (snRNA), and small nucleolar RNA (snoRNA) [6–8]. In higher eukaryotes, Pol II generates long noncoding RNA (lncRNA) and microRNA (miRNA) [9, 10]. Pol II also transcribes cryptic unstable transcripts (CUTs) and stable unannotated transcripts (SUTs), which are degraded after synthesis [11–13]. The suppression of CUTs is important to prevent inappropriate transcription within ORFs, to enhance processivity during transcription elongation, and to prevent gene silencing via histone deacetylation [14–18].

**Figure 1:**
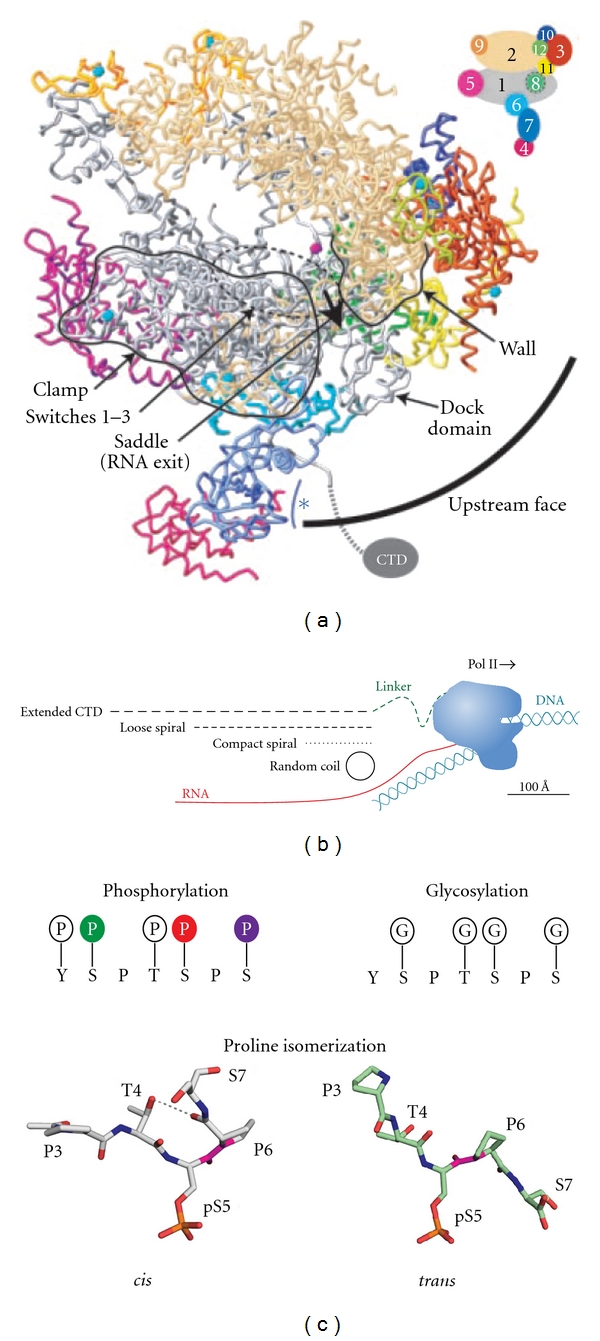
RNA polymerase II structure. (a) Side view of the core Pol II crystal structure containing all twelve subunits and displaying the RNA exit channel (bold arrow) and the positioning of the CTD adapted from Armache et al. [71]. Cartoon in the upper right displays the color coding for the Pol II subunits used in the crystal structure. (b) Illustration of the relative length(s) between the CTD in various conformations and the core Pol II adapted from Meinhart et al. [72]. RNA positioning (red) upon exit of the Pol II and the positioning of the DNA template (blue) upstream and downstream of the core Pol II are also displayed. (c) Known modifications possible on the Pol II CTD are displayed. Glycosylation and phosphorylation are mutually exclusive modifications. Structural images of a heptad repeat in the *cis- *and *trans-*conformation are also shown [73–75]. G: *β*-O-linked N-acetylglucosamine [76]; P: O-linked phosphate.

**Figure 2:**
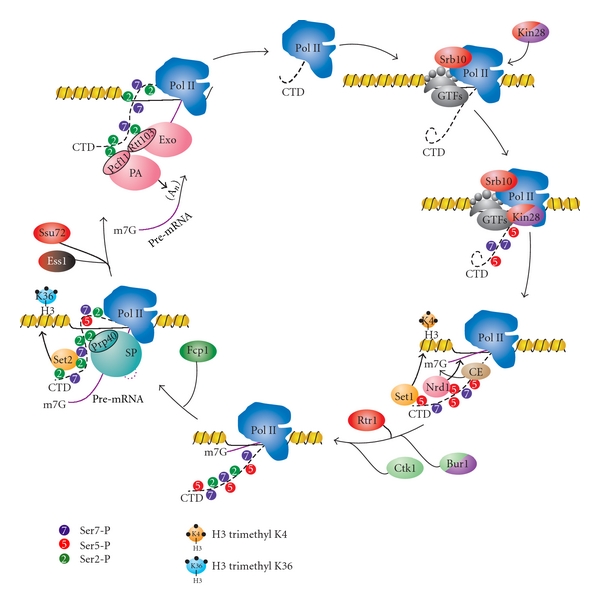
The primary components of the RNA biogenesis machinery and their interactions with the RNA polymerase II C-terminal domain (CTD). Briefly, hypophosphorylated Pol II assembles at the preinitiation complex (PIC) with the Mediator and general transcription factors (GTFs), with TFIIH associating last. The TFIIH-associated kinase Kin28 phosphorylates Ser5 (shown in red) and Ser7 (shown in purple) on the CTD. Mediator-associated kinase Srb10 also contributes to the phosphorylation of Ser5-P. This mark enables promoter release and mediates interactions with the capping enzyme (CE) complex, Nrd1 component of termination machinery, and Set1 histone methyltransferase, which places trimethyl marks on histone H3K4. The Ser5-P mark also facilitates recruitment of Bur1 kinase. Bur1 places initial Ser2-P marks, which facilitate recruitment of Ctk1 kinase, and continues to replenish Ser7-P marks during elongation. Ctk1 is the primary Ser2 kinase, and its phosphorylation recruits splicing machinery (SP) through Prp40, as well as Set2 histone methyltransferase, which places di- and trimethyl marks on histone H3K36. Cleavage and polyadenylation (PA) machinery are recruited through many factors associating with the CTD. One of the factors, Pcf11, binds cooperatively to Ser2-P with Rtt103. The exonuclease complex (Exo) is also recruited through interaction between CTD and Rtt103 and through cooperative interaction between Rtt103 and Pcf11. Finally, the hypophosphorylated CTD is regenerated through three CTD phosphatases. Ser2-P is removed by the phosphatase Fcp1, while two phosphatases, Rtr1 and Ssu72, combine to remove Ser5-P marks during elongation and at termination, respectively. Upon de-phosphorylation, Pol II is released with the assistance of a mechanism involving Pcf11 and can begin another cycle of transcription.

Of the twelve Pol II subunits, five are common between the three polymerases [1, 19–21]. It is believed that the specific functions attributed to each polymerase arise from the combined action of remaining nonidentical subunits and other factors that associate with them. An especially unique feature of Pol II is the carboxy-terminal domain (CTD) of its large subunit Rpb1 (Figure 1(a)). The CTD serves as the primary point of contact for a wide variety of molecular machines involved in RNA biogenesis during the transcription cycle (reviewed in [8, 22–32]). This domain consists of a highly conserved heptapeptide repeat: Y_1_S_2_P_3_T_4_S_5_P_6_S_7_ [33–36]. The number of times this sequence is repeated varies among eukaryotic organisms, ranging from 15 repeats in amoeba, to 26 repeats in the budding yeast *Saccharomyces cerevisiae,* to 52 repeats in humans. When fully extended, the yeast CTD can span a distance of up to 650 Å, over 4 times the diameter of the core polymerase (Figure 1(b)) [24, 34, 35]. The ability of this repetitive sequence to interact with a wide range of nuclear factors stems from the dynamic plasticity of its structure and the diversity of binding surfaces generated by the multitude of post-translational modifications it can accommodate. Tyrosine, threonine, and three serines can all be phosphorylated, the threonine and serine can be glycosylated, and the prolines can undergo isomerization (Figure 1(c)) [27, 37, 38]. In humans, CTD repeats further away from core Pol II bear noncanonical repeats that can be methylated [39]. Taken together, at least 10^59^ unique modification patterns can occur on the CTD. The combinatorial nature of these modifications, which is reminiscent of the histone code, led to the hypothesis of a CTD code, where the patterns of modifications are read by the transcriptional machinery and these patterns dictate the association or disassociation of complexes [40, 41]. To date, much effort has been made towards characterizing these modifications and understanding the interactions between the CTD and components of various protein machines that play a role in RNA biogenesis. Our current knowledge of the integration of these events by Pol II CTD is summarized in Figure 2, and the known yeast CTD-interacting factors are displayed in Table 1. The focus of this paper is to highlight the recent advances in our understanding of the role of CTD in the early stages of the Pol II transcription cycle, expand on the concept of the CTD code hypothesis, and address the current questions and challenges within the field.

### 1.1. RNA Pol II Transcription Cycle

#### 1.1.1. Transcription Initiation

**Figure 3:**
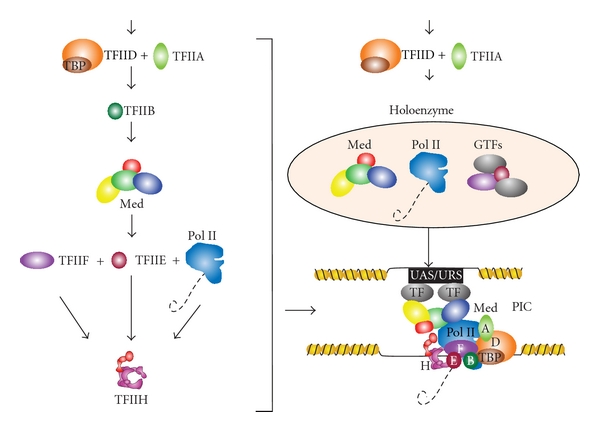
Recruitment and composition of PIC components. Sequential recruitment of the Mediator complex, GTFs, and Pol II (left) or the recruitment of the Pol II holoenzyme (top right), which assembles the pre-initiation complex (PIC) at promoters (bottom right).

Initiation of transcription begins with the recruitment of gene-specific transcription factors (TFs), general transcription factors (GTFs), the Mediator complex, and Pol II. These factors self-assemble into a pre-initiation complex (PIC) at the promoters of Pol II-transcribed genes [29, 32]. Recognition of the promoter is only partially understood, but it is believed to occur via the recognition of the various *cis*-elements in the promoter region, such as the TATA box. Binding generally occurs within upstream nucleosome-free regions—the DNA centered over promoters flanked by well-positioned nucleosomes [42–45]. There are two main models for how these factors assemble at this region: the sequential model and the holoenzyme model (Figure 3). In both models, TFs first bind at the upstream activating/repressing sequences (UAS/URS) and recruit the transcriptional machinery. In the sequential model, TBP/TFIID/SAGA assembly at the promoter is accompanied by TFIIA, followed by TFIIB [46, 47]. Then, the Mediator complex arrives, connecting the PIC to transcription factors assembled at the UAS/URS [48–51]. This massive complex consists of three large modules known as the head, middle, and tail and an additional kinase module containing a cyclin-dependent kinase (Srb10 in yeast, Cdk8 in metazoans) [52–57]. The Mediator complex is important for basal transcription and plays a central role in facilitating communication between transcription factors bound to regulatory elements and the PIC [49–51, 56–60]. However, there are studies that suggest the Mediator is not present at most genes, and it only associates with a few UAS/URS in an activator- and stress-specific manner [61, 62]. Pol II is then recruited, followed by the last GTF, TFIIH, which is brought to the PIC by TFIIE [63]. It is possible that several pathways of ordered recruitment exist for GTFs. Other components, including Pol II, TFIIE, and TFIIH, may be recruited via interactions with the Mediator [64]. The holoenzyme model originated from the observation that Srb proteins, which are components of the Mediator, are tightly associated with core Pol II in the absence of DNA [65]. In this model, Pol II is associated with the Mediator and other general transcription factors as a massive holoenzyme supercomplex that is recruited immediately after TBP binds [66–68]. These complexes have been identified in yeast and mammalian systems [69]. Importantly, Pol II is fully able to activate transcription upon arrival in this state [68, 70].

Two complexes of the PIC, TFIIH and the Mediator, contain important kinases that phosphorylate the CTD. TFIIH is a ten-subunit complex containing two helicases, an ATPase, a ubiquitin ligase, a neddylation regulator, and a cyclin-dependent kinase (Kin28 in yeast, Cdk7 in metazoans) [111–118]. Both Kin28/Cdk7 and Srb10/Cdk8 have been shown to phosphorylate Ser5 (Ser5-P) *in vivo*, with Kin28/Cdk7 being the dominant kinase [24, 113, 119–124]. The 5′ enriched Ser5-P mark has been linked to a variety of chromatin-modifying and RNA processing events.

#### 1.1.2. Transcription Elongation

Phosphorylation of Ser5 is involved in coordinating the placement of several key posttranslational modifications on chromatin that constitute the histone code [41] (reviewed in [125–127]). The structural properties of chromatin, such as the +1 nucleosome that resides immediately after gene promoters, are thought to provide a significant physical barrier to transcription. This barrier is weakened or removed through the combined action of posttranslational modifications on the flexible histone tails and chromatin remodeling complexes [127]. In this context, the Ser5-P mark recruits the yeast histone methyltransferase Set1. Trimethylation of histone H3K4 by Set1 and subsequent trimethylation of H3K79 by Dot1 are frequently associated with active transcription and have a reciprocal effect on H3K14 acetylation by SAGA and NuA3 [28, 86, 128, 129]. Ser5-P also recruits the histone deacetylase complexes Set3 and Rpd3C(S) [87], which are important for suppressing CUT initiation at promoters [87, 88].

An especially important role of Ser5-P is the recruitment of the capping enzyme complex. The capping complex places the m^7^G cap on the nascent transcript as it exits the core polymerase, stabilizing the mRNA by preventing its degradation by 5′-3′ exonucleases. The CTD repeats proximal to the core Pol II are ideally placed near the RNA exit tunnel to facilitate this capping reaction [130, 131]. The guanylyltransferase (Ceg1 in *S. cerevisiae*) and possibly the methyltransferase (Abd1 in *cerevisiae*) directly interact with both the Ser5-P and the core polymerase [80–85, 132, 133]. Although the recognition of the CTD is structurally different between yeast and mammalian capping enzymes, both complexes require Ser5-P for binding [81, 131]. A parallel line of experiments showed that inhibition of Kin28 kinase activity using a small-molecule inhibitor leads to a severe reduction in Ser5-P and 5′-capping of transcripts at gene promoters [134, 135]. In agreement with this, tethering the mammalian capping enzyme to the CTD rescues the null Ser5 to alanine mutants in the fission yeast *Schizosaccharomyces pombe* [136]. Interestingly, inactivation of Kin28 does not eliminate transcription: neither steady-state mRNA levels nor the ability to initiate transcription at the inducible *GAL1* gene is significantly compromised by the inhibition [135]. A subsequent study using the same chemical inhibition system confirmed the earlier observations but incorrectly attributed small differences in transcript levels to inappropriate normalization of earlier microarray data [137]. No such global normalization was performed by Kanin et al. [135] and it is unclear why the subsequent study [137] made the unsubstantiated and erroneous claim that the data was treated incorrectly. Kanin et al. were quite cognizant of the consequences of inhibiting an enzyme that could have a role in global transcription. Moreover, quantitative PCR and northern blot assays, experiments that were not reliant on microarray normalization, showed little difference in expression (Hein and Ansari, 2007, unpublished data) [135]. These results strongly support the conclusion that inactivating Kin28 does not significantly impact global transcription. It is important to note that these studies only focused on chemical inhibition of Kin28 and that the inhibition is not an “all or none” phenomenon due to equilibrium binding of the small molecule to the kinase; it is possible that extremely low levels of Ser5 phosphorylation, by either Srb10 or residual Kin28, suffice for transcription initiation. Importantly, chemical inhibition of both Kin28 and Srb10 shows a drop in Pol II across the ORF, supporting the model where Ser5-P may help in promoter clearance [138].

We and others have recently demonstrated that Kin28/Cdk7 is also the primary kinase that phosphorylates Ser7 (Ser7-P) [139–141]. The phosphorylation occurs at protein-coding and noncoding genes and seems to be Mediator dependent [142]. Cyclin-dependent kinases are thought to prefer a substrate bearing Ser-Pro rather than Ser-Tyr dipeptides [143]. Additionally, while Kin28 has been localized to promoters [83], Ser7-P marks were thought to be found only at non-coding genes and at the 3′ end of protein coding genes [144, 145]. The role of Ser7-P at promoters remains an active area of investigation.

**Figure 4:**
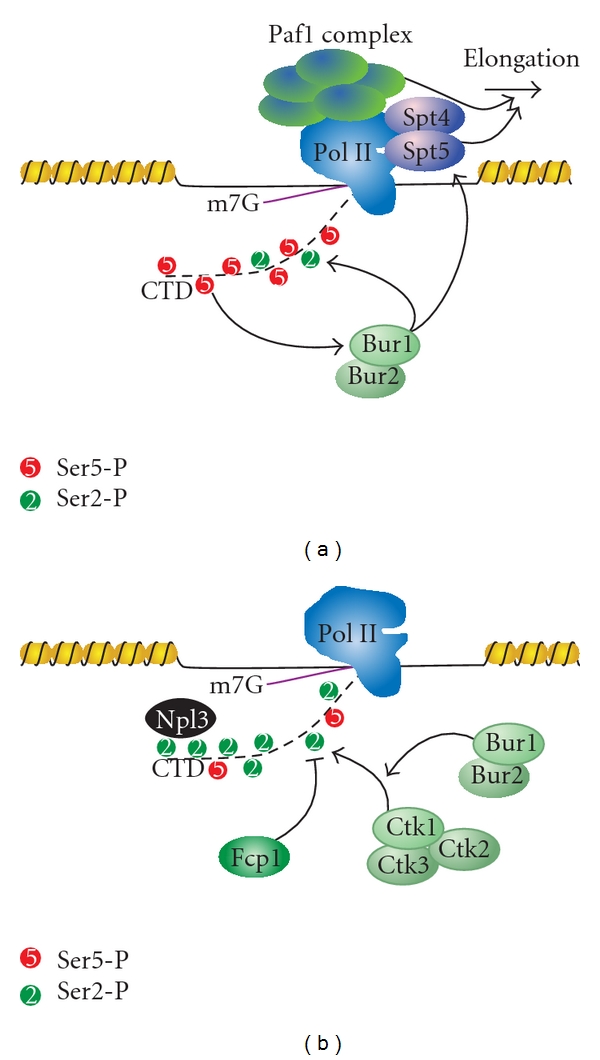
Bur1 phosphorylation of the CTD facilitates the transition from initiation to elongation. (a) Ser5-P enhances recruitment and subsequent phosphorylation of Ser2 by Bur1. Bur1 also phosphorylates Spt5, which acts with the Paf1 complex to promote elongation. (b) CTD phosphorylation by Bur1 enhances the activity of Ctk1 on Ser2. The majority of the Ser2-P is maintained by competition between phosphorylation by Ctk1 and dephosphorylation by Fcp1. This increase in Ser2-P facilitates recruitment of many Ser2-P-binding proteins, such as Npl3.

Following promoter clearance, transcription initiation factors are exchanged for transcription elongation factors required for RNA processing, passage through chromatin, and suppressing cryptic transcripts. In budding yeast, this exchange occurs immediately after the +1 nucleosome [146]. The association of these elongation factors, which include Paf1, Spt16, Spt4, Spt5, Spt6, Spn1, and Elf1, occurs concurrently on all Pol II genes and is independent of gene length, type, or expression [146]. The recruitment of these factors is essential for transcription processivity (Spt4/5) [147–149], histone regulation (Spt6/16, Spn1, Elf1) [150–156], and gene activation/3′ processing (Paf1) [157]. Similarly, mammalian P-TEFb complex is recruited to Pol II at this stage of transcription [158–161]. This complex contains a cyclin-dependent kinase (Cdk9) that phosphorylates the DRB-sensitivity-inducing factor (DSIF), which allows Pol II to overcome the promoter-proximal pausing induced by the negative elongation factor (NELF) complex [23, 159]. It is unclear if promoter-proximal pausing occurs in yeast, but it is known that Bur1 (the yeast homolog of Cdk9) promotes elongation through post-translational modification of Spt5 (DSIF) (Figure 4(a)) [162]. Bur1 also improves transcription elongation through the recruitment of histone-modifying enzymes and the phosphorylation of CTD. Bur1 activity promotes the ubiquitylation of H2BK123 by the ubiquitin conjugating enzyme Rad6 and Bre1 [129, 163]. H2BK123Ub promotes Set1 trimethylation of histone H3K4 and subsequent trimethylation of H3K79, both of which are important for transcription activation [28, 86, 128, 129]. Bur1 also promotes transcription elongation by coupling promoter-proximal CTD modifications with promoter-distal marks. Bur1 is recruited to the transcription complex by the Ser5-P marks placed at the promoter. It then phosphorylates Ser2 (Ser2-P), priming the CTD for the recruitment of Ctk1 (Cdk12), the major Ser2 kinase [164]. Initial CTD phosphorylation also increases the activity of Ctk1, thereby coupling sequential CTD modifications (Figure 4(b)) [23, 159, 165, 166]. Interestingly, Bur1 travels with Pol II and phosphorylates Ser7-P. Although the exact role of this modification is unclear, it is likely a mark that promotes elongation, as genes with uniformly high levels of Ser7-P are transcribed at significantly higher levels [138].

Most Ser5-P marks are removed near the +1 nucleosome through the action of the newly characterized CTD phosphatase Rtr1 [167]. This phosphatase has been shown to specifically remove Ser5-P marks immediately after promoter clearance. The Ser2-P phosphatase Fcp1 also associates during elongation, but Ser2-P levels remain high across the transcript due to the opposing action of the Ser2-P kinase Ctk1 [168, 169]. It is thought that the Ubp8 component of SAGA travels with Pol II and promotes deubiquitylation of H2BK123Ub [170], which allows the association of Ctk1 and subsequent phosphorylation of Ser2 on the CTD [171].

Ser2-P is critically important for the interaction between the CTD and many histone modifying and RNA processing machines [75, 83, 132, 172–178]. Increasing levels of Ser2-P, in combination with the residual Ser5-P, promote the recruitment of the Set2 methyltransferase, which catalyzes the formation of H3K36me2 and H3K36me3 [95, 96, 179–181]. This leads to the recruitment of the histone deacetylase complex Rpd3C(S) and the removal of acetylation from histones H3 and H4, thereby resetting the transcription state of the nucleosomes and repressing cryptic transcription within ORFs [87, 182, 183]. Ser2-P is involved in the co-transcriptional and posttranscriptional processing of RNA. Cotranscriptional processing of introns via splicing involves the yeast protein Prp40, which preferentially associates with Ser2-P/Ser5-P marked CTD [97]. Ser2-P is also bound by the SR-like (serine/arginine rich) protein Npl3, which functions in elongation, 3′-end processing, hnRNP formation, and mRNA export [184–187]. Finally, increasing levels of Ser2-P, coupled with depletion of Ser5-P, leads to the recruitment of the termination and polyadenylation machinery (discussed below).

#### 1.1.3. Transcription Termination

The role of CTD modifications in orchestrating transcription termination is better described in recent reviews [31, 188]. In essence, two models have been proposed to explain how Pol II termination occurs, with the emerging view being that it is likely a combination of the two models that best describes the mechanism. The first model, known as the “allosteric” or “antiterminator” model, proposes that transcription through the polyadenylation site leads to an exchange of elongation factors for termination factors, resulting in a conformational change of the elongation complex. Indeed, this model is supported by chromatin immunoprecipitation (ChIP) data of elongation factor exchange at the 3′ end of genes [146, 189]. The second model, known as the “torpedo” model, postulates that cleavage of the transcript at the cleavage and polyadenylation site (CPS) creates an entry site for the 5′-3′ exonuclease Rat1 (Xrn2 in mammals), which degrades the 3′ RNA and promotes Pol II release by “torpedoing” the complex [189–191]. In this model, recruitment of Rat1 is likely to be indirect, possibly through its partner Rtt103. Rtt103 has been shown to bind Ser2-P in a cooperative manner with Pcf11 [192], an essential component of the cleavage factor IA (CFIA) complex that also promotes Pol II release [193]. Interestingly, ChIP data shows Pcf11 at both protein-coding and noncoding genes, and mutating Pcf11 results in terminator read-through due to inefficient cleavage at both gene classes [75, 174, 193–196]. Pcf11 may play an important role in both the termination and processing of protein-coding and non-coding genes.

**Figure 5:**
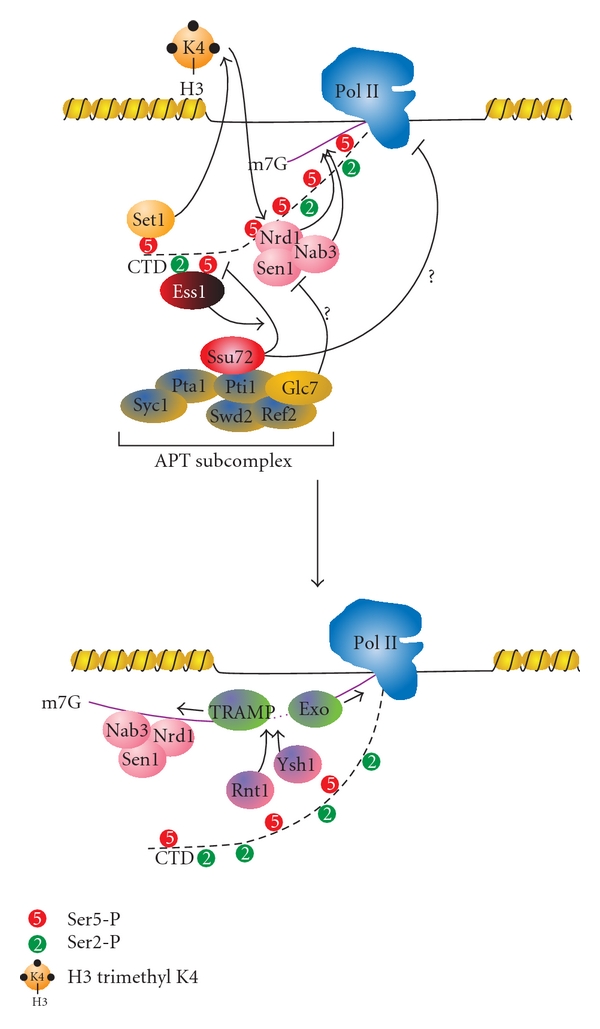
Nrd1-dependent termination pathway. The Nrd1-Nab3-Sen1 complex is recruited via interaction between Nrd1 and Ser5-P. This recruitment is facilitated by H3K4me3, which is placed by the Set1 histone methyltransferase. The mechanisms by which the Ssu72 and Glc7 phosphatases promote termination are still unclear, but it may be that the dephosphorylation of Sen1 by Glc7 and of the CTD by Ssu72 causes the polymerase to pause, and allowing the termination machinery to associate. During elongation, both Nrd1 and Nab3 scan the nascent RNA for their preferred sequences (see text for details). Upon finding their concensus sequences, Nrd1-Nab3-Sen1 complex is able to be associated with the RNA. The endonucleases Rnt1 and Ysh1 may contribute to the cleavage of the RNA, which is followed by 3′-5′ trimming the transcript by the TRAMP complex and by the degradation of the remaining RNA exiting Pol II by the 5′-3′ exonuclease Rat1 (Exo).

**Figure 6:**
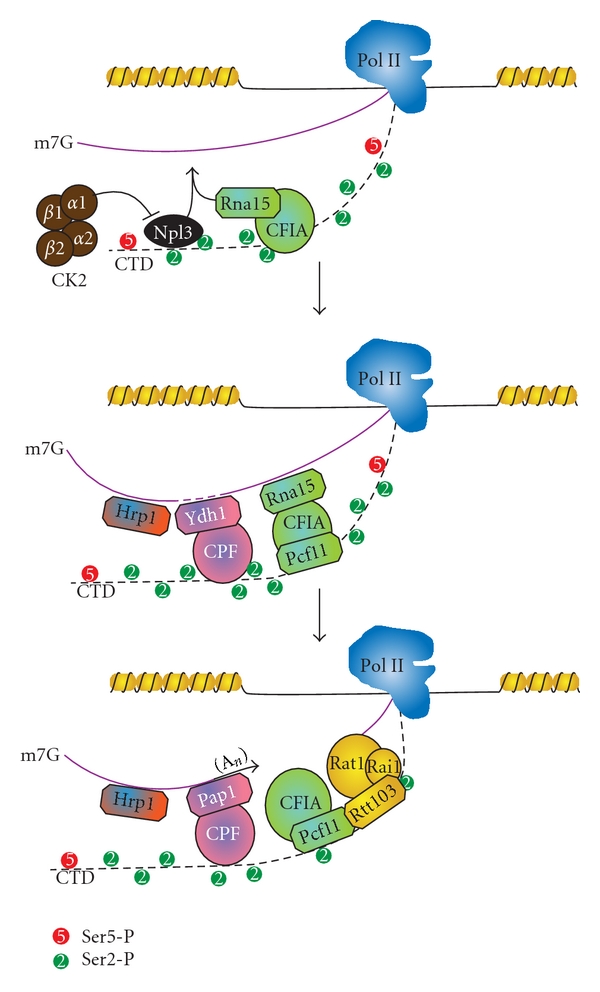
The mRNA termination pathway. Rna15 competes with Npl3 for binding to the nascent RNA. CK2 phosphorylates Npl3, allowing Rna15 to find its preferred binding site (an A/U-rich region) in the RNA. The CPF and CFIA components assemble through interactions with the CTD and the Yth1 component of CPF cleaves the nascent RNA at the polyadenylation site, followed by polyadenylation by Pap1. Then the Rat1 exonuclease complex associates via cooperative interaction between Pcf11 and Rtt103 and leads to termination and dissociation of Pol II.

Processing of Pol II transcripts occurs via one of two distinct, gene class-specific pathways in yeast. Many small mRNAs (<550 bp), CUTs, snRNA, and snoRNAs (non-coding genes) are processed via the Nrd1-Nab3 pathway (Figure 5), while longer mRNAs (protein-coding genes) are processed in a polyadenylation-dependent process (Figure 6) [8, 11, 12, 27, 31, 178, 195, 197–199]. The decision to proceed down a certain processing path is modulated by the phosphorylation state of the CTD. Nrd1 preferentially associates with Ser5-P, and its recruitment is also enhanced via histone H3K4 trimethylation by Set1 [90, 200]. Nrd1 and Nab3 scan the nascent RNA for specific sequence elements (GUAA/G or UGGA for Nrd1, and UCUU or CUUG for Nab3) as it exits the core polymerase [90, 199, 201–207]. The helicase Sen1 (senataxin in humans), which exists in complex with Nrd1 and Nab3, resolves the DNA:RNA hybrids known as R-loops that form between the template DNA and the nascent RNA, keeping the specific sequence elements exposed and preserving genomic stability [208–210]. The involvement of Sen1 is dependent on the phosphatase Glc7, which dephosphorylates Sen1 and is essential for the proper termination of snRNA and snoRNA transcripts [211]. Upon detecting its consensus sequence elements, the Nrd1 complex and the Rnt1 endonuclease cleave these short transcripts [195, 212–214], which are then trimmed at the 3′ end by the TRAMP complex and the exosome [6, 215–217]. Nrd1 then disengages from the transcription complex, with help from antagonizing Ser2-P marks [198]. Unlike snRNA/snoRNAs, which have protective structural elements in the RNA, Nrd1-terminated CUTs have no protective elements at their 3′ ends and are thus fully degraded by TRAMP after cleavage [8, 11, 12]. Nrd1 has been mapped to the 5′ end of transcribed regions, but a recent study has demonstrated that Nrd1 occupancy is maintained across the open reading frame of genes [196]. Although no homolog of Nrd1 has been found in mammalian cells, the Integrator complex that is involved in 3′ processing of snRNA transcripts is recruited by Ser7-P [218]. The association of this complex with Ser7-P CTD was demonstrated by the abolishment of this interaction upon mutation of Ser7 to alanine [145]. Subsequent analysis using a panel of CTD peptides determined that the Integrator prefers to bind a diphosphorylated CTD substrate spanning two heptad repeats in the S7-P-S2-P conformation [219]. It is possible that Ser7-P may serve as a similar scaffold for snRNA and snoRNA processing machinery in yeast.

The second pathway, used for the processing of most mRNA transcripts, involves the cleavage and polyadenylation factor (CPF) complex, cleavage factor IA and IB (CFIA and CFIB) complexes, and the exosome (Figure 6) [31, 195, 197]. Many of the termination and 3′ processing factors involved in this process are known to preferentially associate with Ser2-P or Ser2-P/Ser5-P enriched CTD including: Npl3, Rtt103, Rna14, Rna15, Ydh1, Yhh1, Pta1, and Pcf11. In this pathway, Rna15 competes with Npl3 for recognition of a UA-rich site in the nascent RNA [98, 187]. This competition is removed upon phosphorylation of Npl3 by casein kinase 2 (CK2) [98]. Rna15 can then bind the nascent RNA and promote endonucleolytic cleavage followed by polyadenylation by the polyadenylate polymerase (Pap1) [197, 220]. Polyadenylation-binding proteins (PAB) then protect the mature transcript from exonucleolytic degradation (Figure 6) [221].

In both pathways, the CTD is hypophosphorylated by the combined action of two essential phosphatases at the end of transcription: Ssu72 and Fcp1. Ssu72 is a member of the Associated with Pta1 (APT) complex, which is present at both gene classes and is involved in 3′ processing of non-coding RNAs [222]. As such, Ssu72 is primarily localized at the 3′ end of transcripts [222], although there is one instance in which it has been found at promoters [223]. Temperature-sensitive mutants of Ssu72 exhibit read-through at both protein-coding and non-coding transcripts [224]. Ssu72 is the primary Ser5-P phosphatase [225], and its phosphatase activity is enhanced by the prolyl isomerase Ess1/Pin1 and by interacting with Pta1/symplekin [226–228]. Recently, crystal structures have shed light on the mechanism of Ssu72: the phosphatase binds to Ser5-P only when the adjacent Pro6 is in the *cis*-conformation [73, 74]. In contrast to Ssu72, Fcp1 associates with TFIIF during transcription and is found across the entire transcribed region [168, 169, 229, 230]. Although it has Ser5-P and Ser2-P phosphatase activity *in vitro*, Fcp1 is considered a Ser2-P-specific phosphatase *in vivo* [231, 232]. Fcp1 activity is enhanced upon phosphorylation of Fcp1 by CK2 [233]. Defects in Fcp1 also result in transcription read-through at Nrd1-dependent transcripts [198]. Though it is unclear which phosphatase removes Ser7-P, new data from our lab suggest that Ssu72 may be the phosphatase that removes Ser7-P at both the 5′ and 3′ ends of genes [234]. Removal of this mark may be even more important than its placement as mutation of Ser7 to alanine slows growth while mutating Ser7 to the phosphomimic glutamate is lethal [144].

Global dephosphorylation of the CTD facilitates the release of Pol II from DNA, which can then recycle to promoters for the next cycle of transcription [224, 235, 236]. It has been proposed that transcription termination and subsequent dephosphorylation of the CTD is coupled to transcription reinitiation through gene looping, by which the promoter and terminator regions are brought together, allowing Pol II to associate more rapidly with the PIC [237, 238]. Intriguingly, Ssu72 and the GTF TFIIB have been shown to be essential in gene looping [223, 239]. Taken together, the phosphorylation and dephosphorylation of the CTD is intimately involved in every phase of transcription, from initiation, to elongation, to termination, and possibly reinitiation.

#### 1.1.4. Other Regulatory Roles of the CTD

**Figure 7:**
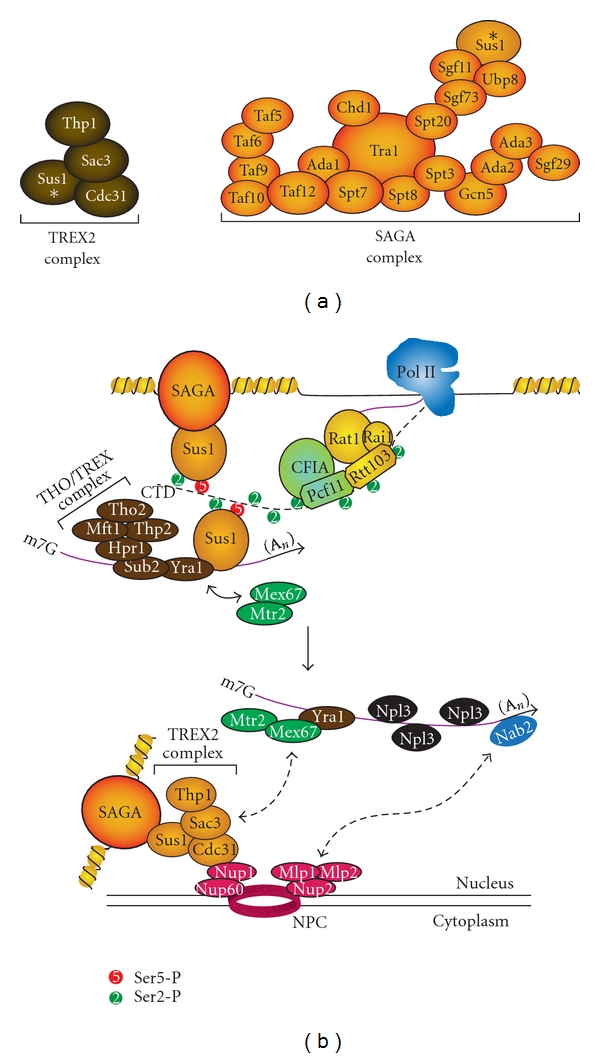
Sus1 in TREX2 and SAGA complexes coordinates mRNA export. (a) Subunit compositions of TREX2 and SAGA complexes are shown, highlighting Sus1 (asterisk). (b) mRNA export coordinated by Sus1. Sus1 binds Ser2-P and Ser2-P/Ser5-P CTD, connecting the CTD to the SAGA histone acetyltransferase complex. Sus1 also interacts with Yra1 component of the THO/TREX complex on the RNA. Mex67-Mtr2 are recruited by interaction with Yra1 and help form the export-competent mRNP. At the nuclear pore complex (NPC), Mlp1-Mlp2 interact with the polyA mRNA-binding protein Nab2 and Mex67 interacts with Sac3 of the TREX2 complex. This interaction brings the export-competent mRNP to the NPC in preparation for export to the cytoplasm. Sus1 is a component of both TREX2 and SAGA and serves to tether actively transcribed gene promoters to the NPC.

In addition to its many roles in transcription initiation, elongation, and termination, the CTD has been implicated in a variety of transcription-extrinsic processes, such as mRNA export and stress response. mRNA export (reviewed in [240–242]) requires the packaging of the mRNA into export-competent messenger ribonucleoprotein (mRNP) via association with the Mex67:Mtr2 heterodimer [243]. This heterodimer is brought to the mRNA by Yra1 and Sub2, components of the THO subunit of the TREX1 complex [242]. The process of mRNP export is coordinated by the protein Sus1. This central protein directly interacts with Ser5-P and Ser2-P/Ser5-P of the CTD, Ub8 subunit of the SAGA complex, Yra1 subunit of the TREX1 complex, and Sac3 subunit of the TREX2 complex at the nuclear pore (Figure 7) [106, 244].

The CTD is also involved in stress response. The ubiquitin ligase Rsp5 binds the CTD and ubiquitylates Pol II in response to DNA damage [245, 246]. Similarly, UV-induced DNA damage in mammalian fibroblasts results in hyperphosphorylation of the CTD by the mammalian positive transcription elongation factor b (P-TEFb), which then regulates Pol II ubiquitylation and subsequent degradation [247]. Under conditions not well understood, Ser5-P can also recruit the Asr1 ubiquitin ligase, which ubiquitylates the Rpb1 and Rpb2 subunits of Pol II. This ubiquitylation promotes ejection of the Rpb4/7 heterodimer from the core polymerase and inactivates Pol II, which may provide a mechanism for stopping polymerases engaged in abortive or cryptic transcription [92].

## 2. The CTD Code Controversy: Is It a Code?

The concept of the CTD code was first proposed due to the enormous amount of information that can be encoded via post-translational modification of the CTD repeats [40, 248]. The code would coordinate the assembly of complexes that “read, write, and erase” the code during transcription. Historically, the Ser5-P and Ser2-P marks have been the best characterized, with the canonical distribution of Ser5-P being enriched at the 5′ end of genes and Ser2-P enriched towards the 3′ end. Recently, our lab and several others have been able to map the phospho-CTD occupancy profiles across the yeast genome [135, 138, 139, 146, 196]. There are interesting discrepancies between the observations made by various groups. For example, Mayer et al. find the canonical profile to be present at every gene with Ser7-P profiles overlapping with Ser5-P [146], while we find clusters of genes with noncanonical CTD profiles for Ser2-P, Ser5-P, and Ser7-P [138]. We observe gene-specific phosphorylation profiles, with Ser2-P levels being significantly lower at non-coding genes and Ser7-P profiles diverging from Ser5-P profiles only at protein-coding genes. The distinct patterns of CTD marks at these two gene classes reflect the different mechanisms of transcription termination and 3′ end processing machinery that act on these two classes of RNA. Similarly, Kim et al. also observe differences in phospho-CTD profiles at snoRNAs and at introns [196]. However, the positions of the Ser5-P and Ser7-P peaks in Kim et al. are offset from Tietjen et al. and Mayer et al. Importantly, all three genome-wide analyses reveal an unexpected degree of cooccurrence of CTD marks, suggesting a bivalent or even multivalent mode of recognition by docking partners. In support of this idea, the Set2 histone methyltransferase and the Integrator complex have been shown to prefer a bivalent mark rather than a single phosphorylated residue [95, 96, 219].

In addition to the various phosphorylation marks, the isomerization state of the CTD also contributes to the complexity of the code. For example, Pcf11 binds the CTD in the *trans-*conformation while Ssu72 prefers a *cis*-CTD as substrate [73–75]. Many in the transcription field have made the argument that the CTD code is not a true code because it does not convey biological information via a rigorous decoding key. However, research in the last several years has demonstrated that specific phosphorylation marks and proline isomerization are important for conveying information from *cis-*elements encountered by Pol II to the protein complexes necessary for successful progression through the transcription cycle. Further investigation into the mechanism of this information transfer will resolve the controversy over the existence of a CTD code.

## 3. Future Directions

Extraordinarily rapid progress has been made over the last several years in the field of CTD research; however, many important questions remain unanswered. Although the profiles of Ser7-P have been mapped and several of its kinases discovered, its function at protein coding genes remains unclear. Additionally, most of the kinases identified are established members of the transcription initiation or elongation complexes. One could expect to find new enzymes that could modulate the CTD in response to signals, as post-translational modifications are often used as a mechanism for cells to respond to external stimuli. The recent discovery of Ser7-P at elongating Pol II has also prompted the question of whether Tyr1 and Thr4 phosphorylation (Tyr1-P and Thr4-P) occurs? Tyr1 can be phosphorylated by c-Abl in mammals, but no homolog is present in yeast [249]. In addition, both Tyr1-P and Thr4-P has been detected in *S. pombe* [250]. Interestingly, Tyr1-P and Thr4-P were found in both the hyperphosphorylated and hypophosphorylated states of Pol II, opening the possibility of CTD function independent of transcription. However, neither the profile nor function of these potential modifications have been extensively characterized.

The role of non-canonical residues and their modification states on mammalian CTD remain to be explored. In mammals, the Ser7 residue is only weakly conserved in polymerase-distal repeats of the CTD, often changed to lysine or arginine [144]. Interestingly, Arg1810 of *rpb1* in the human CTD is methylated by the coactivator-associated methyltransferase1 (CARM1) [39]. This methylation occurs prior to both transcription initiation and phosphorylation of Ser2 or Ser5, and mutation of this residue results in the improper expression of a variety of snRNAs and snoRNAs. In addition to methylation, the CTD may also be subject to glycosylation. Recent studies suggest O-GlcNAc are transferred to Ser5 and Ser7 by O-GlcNac transferase and removed by O-GlcNAc aminidase during PIC assembly. This cycling of O-GlcNAc may be important for preventing aberrant CTD phosphorylation by TFIIH [251].

Besides the characterization of novel marks, significant structural challenges remain for understanding the known phosphomarks. One limitation of ChIP is its inability to identify the exact phosphorylation patterns across individual CTD repeats *in vivo* at different points during the transcription cycle. Recent mutational analysis suggests that the minimal functional unit of the CTD consists of three consecutive Ser-Pro dipeptide residues in a S2-S5-S2 configuration [36], but it is unclear if all three serines can be phosphorylated on one functional unit or if phosphorylation alternates between repeats. The lack of positively charged aminoacids makes the phospho-CTD patterns difficult to decipher via mass spectrometry. Additionally, the highly repetitive nature of the CTD makes it difficult to distinguish between the first repeat and the twenty-first. Consequently, the position along the CTD where interacting partners associate remains a mystery. Mutation of Ser2 to glutamate in the core-distal repeats and mutation of Ser5 to glutamate in the core-proximal repeats are lethal [252]. However, this does not directly demonstrate whether the proteins that bind these phosphorylated residues are located at these repeats. Characterizing the phosphorylation patterns and protein occupancies at individual repeats will help determine the existence of a “CTD recognition” code, and this promises to be one of the most exciting and important challenges in the future of CTD research.

## Figures and Tables

**Table 1:** Proteins known to bind RNA polymerase II C-terminal domain in *S. cerevisiae. *

Protein/complex	Role in RNA biogenesis	Phospho-CTD bound	References
TFIIE	Preinitiation complex	Hypophosphorylated CTD	[63, 77]
TFIIF	Preinitiation complex	Hypophosphorylated CTD	[77]
TBP	Preinitiation complex (TFIID)	Hypophosphorylated CTD	[78]
Mediator Complex	Transcription activation/repression	Hypophosphorylated CTD	[48, 79]
Ceg1	Capping	Ser5-P	[80–85]
Abd1	Capping	PCTD	[83]
Set1	Histone methylation	Ser5-P	[86]
Rpd3C(Rco1)	Histone deacetylation	Ser2-P + Ser5-P	[87, 88]
Spt6	Histone chaperone	Ser2-P	[89]
Nrd1	Transcription termination/processing	Ser5-P	[90]
Sen1	Transcription termination/processing	Unknown	[91]
Asr1	Pol II ubiquitylation (Rpb4/7 Ejection)	Ser5-P	[92]
Ess1	Proline isomerase	Ser2-P	[93, 94]
Set2	Histone methylation	Ser2-P + Ser5-P	[95, 96]
Prp40	Splicing	PCTD	[97]
Npl3	Promotes elongation/prevents polyadenylation	Ser2-P	[98]
Pcf11	Cleavage/polyadenylation (CF1A)	Ser2-P	[99, 100]
Rna14	Cleavage/polyadenylation (CF1A)	PCTD	[101]
Rna15	Cleavage/polyadenylation (CF1A)	PCTD	[101]
Ydh1	Cleavage/polyadenylation (CPF)	PCTD	[102]
Yhh1	Cleavage/polyadenylation (CPF)	PCTD	[103]
Pta1	Cleavage/polyadenylation (CPF)	Ser5-P	[104]
Rtt103	5′-3′ Exonuclease (Rat1)	Ser2-P	[105]
Sus1	mRNA export	Ser5-P	[106]
Yra1	mRNA export	Hyperphosphorylated CTD	[107]
Rsp5	Pol II ubiquitylation (DNA damage response)	Ser2-P	[108, 109]
Hrr25	DNA damage repair	PCTD	[24, 110]

CTD-interacting proteins, the processes they are involved in, the phosphorylation state of the CTD with which they associate, and where in the literature the interaction is documented. Ser2-P refers to phosphorylated serine 2, Ser5-P refers to phosphorylated serine 5, and PCTD refers to a mixed phosphorylation state generated by *in vitro* phosphorylation of a CTD peptide with cell extracts. Additional protein-CTD interactions are described [110] but have not been directly tested.
